# Safety and Effectiveness of Intensive Treatments Administered Outside the Intensive Care Unit to Hematological Critically Ill Patients: An Intensive Care without Walls Trial

**DOI:** 10.3390/jcm12196281

**Published:** 2023-09-29

**Authors:** Beatrice Vergnano, Davide Signori, Annalisa Benini, Serena Calcinati, Francesca Bettini, Luisa Verga, Lorenza Maria Borin, Fabrizio Cavalca, Carlo Gambacorti-Passerini, Giacomo Bellani, Giuseppe Foti

**Affiliations:** 1 Department of Anesthesia and Intensive Care Medicine, Asst Monza, San Gerardo Hospital, 20900 Monza, Italy; 2 Department of Medicine and Surgery, University of Milan-Bicocca, Piazza Ateneo Nuovo 1, 20126 Milano, Italy; 3 Department of Hematology, Asst Monza, San Gerardo Hospital, 20900 Monza, Italy

**Keywords:** medical emergency team, ICU admission, CPAP, hematological critically ill patients, prognostication

## Abstract

Historically, the admission of hematological patients in the ICU shortly after the start of a critical illness is associated with better survival rates. Early intensive interventions administered by MET could play a role in the management of hematological critically ill patients, eventually reducing the ICU admission rate. In this retrospective and monocentric study, we evaluate the safety and effectiveness of intensive treatments administered by the MET in a medical ward frame. The administered interventions were mainly helmet CPAP and pharmacological cardiovascular support. Frequent reassessment by the MET at least every 8 to 12 h was guaranteed. We analyzed data from 133 hematological patients who required MET intervention. In-hospital mortality was 38%; mortality does not increase in patients not immediately transferred to the ICU. Only three patients died without a former admission to the ICU; in these cases, mortality was not related to the acute illness. Moreover, 37% of patients overcame the critical episode in the hematological ward. Higher SOFA and MEWS scores were associated with a worse survival rate, while neutropenia and pharmacological immunosuppression were not. The MET approach seems to be safe and effective. SOFA and MEWS were confirmed to be effective tools for prognostication.

## 1. Introduction

Advances in oncology and hematology, with significant progress in chemotherapy regimens or targeted therapies, led to improved survival in patients with cancer. As a result, a growing number of patients are living with active hematological malignancies and are at risk for life-threatening acute illness requiring intensive care unit (ICU) support [1,2].

Within the last 25 years, cancer and ICU treatment have clearly improved, and the survival rate increased from 2.5% [3] to more than 60% in recent studies [2]. Moreover, the most recent literature clarifies that the nature and staging of the underlying neoplastic disease has little impact on mortality after admission to the ICU; in contrast, the acute disease itself and the baseline health status and comorbidities are considered the main predictors of ICU survival [4,5,6,7]. Thus, a consistent number of the “classical” mortality predictors have lost their value, and novel clinical approaches are advocated for the admission of high-risk cancer patients into intensive care [4].

The admission of hematological patients to the ICU shortly after the onset of the critical illness is associated with better survival rates [2,4,5,8]. Despite these data, prompt ICU admission cannot always be achieved, partly because the stigma of a dismal prognosis still accompanies these patients and partly due to triage criteria variability, with the latter being closely related and interdependent with ICU resources availability [6,7]; the critical issue of the allocation of ICU resources has been heavily highlighted during the current pandemic of SARS-CoV-2 [9].

The policy of admission to intensive care of the onco-hematological patient population varies significantly in different centers, which hence report huge variations in the survival rates of these patients [6,10]. In recent years, several algorithms have also been proposed to assist clinicians with this decision making. Triage algorithms and protocols can be useful but are still not able to replace the role of a skilled intensivist’s evaluation, significantly when it relies on a multidisciplinary knowledge setting [11,12,13].

The “critical care without walls” concept was first proposed 20 years ago [14], highlighting the relevance of providing critical care expertise in a specialistic ward setting. The MET (Medical Emergency Team) has a fundamental role in identifying critically ill patients, including those with a hematological disease who are at risk of clinical deterioration, avoiding delays in admission to the ICU [6]. The implementation of pre-ICU systems (MET and its equivalents, defined in the literature as the rapid response team or the critical care outreach team) in the management of hematological critically ill patients has been associated with z reduction in both hospital mortality and cardiac arrest outside the ICU [15,16,17]. Moreover, ICU settings do not always guarantee adequate protective isolation in controlled environments, a factor that has been shown to be effective in limiting infectious complications and even mortality in neutropenic patients [5].

However, the role of MET in the management of hematological patients in non-intensive wards is unclear [6]. In this study, we describe the population treated outside the ICU in the hematology ward with the support of MET, analyzing the intensive interventions that were applied. Our aim was to evaluate the safety and effectiveness of an intensive treatment trial in the medical ward provided by MET on hematological patients who develop a critical illness.

## 2. Materials and Methods

This is an observational retrospective monocentric study. Patients were enrolled in San Gerardo hospital, Monza, Italy. Data were collected between January 2015 and December 2019 in a local online registry and analyzed after local ethics committee approval (protocol number 357). Patients’ consent was waived.

Our MET was established in 1997. Its staff is composed of an intensivist physician, an ICU nurse, and an intensive care resident. The team is available 24 h/day and 7 days/week and can be activated by physicians for the management of intra-hospital critical issues and emergencies. In our hospital, the MET also guarantees the automatic follow-up of patients considered at high risk of worsening but still not immediately requiring ICU access. Every patient for which the MET is activated undergoes a multidisciplinary evaluation (generally composed of the hematologist and the intensivist) that leads either to immediate ICU admission or, alternatively, to an intensive treatment upgrade in the hematological ward. The latter is conducted as a short-lasting trial, consisting of helmet CPAP and/or pharmacological cardiovascular support (vasopressors and/or inotropes). Frequent reassessment by the MET at least every 8 to 12 h is guaranteed. This system requires that MET staff are exclusively dedicated to this specific service.

The study was conducted in the hematology ward, which is a high-dependency unit with a nurse-to-patient ratio of 1:4. The ward includes a transplant unit; a Chimeric Antigen Receptor T cell therapies (CAR-T) administration protocol is also active. At least one hematologist is always present in the ward 24 h/day.

### 2.1. Inclusion Criteria

We considered eligible hematological adult patients (age ≥ 18 years) who were referred to the MET for acute clinical deterioration.

### 2.2. Exclusion Criteria

We excluded patients who were not eligible for the ICU treatment due to their clinical condition and poor prognosis defined by a multidisciplinary team composed of hematologists and intensivists. We also excluded those patients who immediately died after the MET referral and for which the MET was alerted for the first time for a cardiac arrest.

### 2.3. Data Collection

Data were collected from electronic patient records and entered, anonymously, into a securely stored database.

We collected demographics, the age-adjusted Charlson comorbidity index (CCI) and Eastern Cooperative Oncology Group (ECOG) scale, the Sequential Organ Failure Assessment (SOFA) score, the Modified Early Warning Score (MEWS) for clinical deterioration, the timing of symptoms onset, and MET referral. Vital parameters (SpO2, arterial pressure, respiratory rate, and mental status), PaO_2_/FiO_2_, vasopressors, and CPAP supports were recorded in the first and last MET evaluations. We also collected data on the hematological baseline condition (diagnosis, disease state, eventual bone marrow transplant and active graft-vs.-host disease, neutropenia, ongoing chemotherapy, and immunosuppression).

### 2.4. Statistical Analysis

Statistical analyses and graphs were performed with IBM SPSS Statistic v. 27.

Continuous variables are summarized as mean values with standard deviations or median values and the interquartile range for normal and non-normal distributions, respectively. Categorical variables were summarized as counts and percentages.

Population characteristic comparison between the control group and the steroid group was performed with an independent-sample T-test for continuous variables and the Chi-Square statistic for categorical variables.

## 3. Results

**Figure 1:**
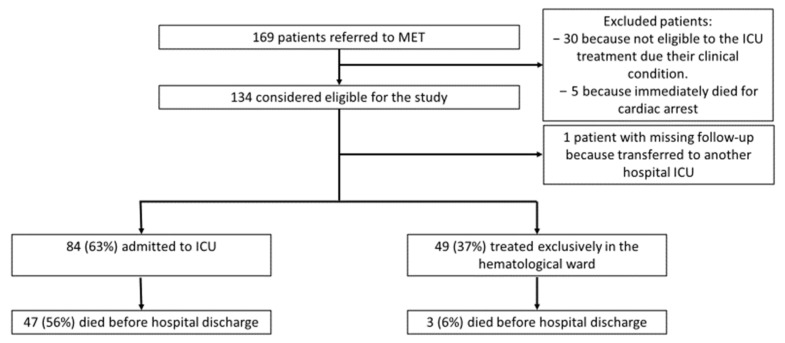
Enrollment, exclusion, and patient distribution in the primary analysis.

Overall, between 14th January 2015 and 30th December 2019, our MET was alerted for 169 hematological patients who had a critical condition according to the attending hematologist. We excluded from the analysis 30 patients who were not considered eligible for ICU treatment due to their clinical condition after a multidisciplinary consensus; also, five patients were excluded because they immediately died at the first MET referral after a failed cardio-pulmonary resuscitation for cardiac arrest. In total, 134 patients were considered eligible for the study. We analyzed data from 133 patients because we missed the follow-up of a patient transferred to an ICU in another hospital. Of these, 84 (63%) were admitted to the ICU, while 49 (37%) were treated exclusively in the hematological ward (Figure 1). Among the patients who were admitted to the ICU, twenty-nine died in the ICU and eighteen died in the hematological ward after ICU discharge, without being further referred to the MET (total ICU mortality of 56%). Three patients died in the hematological medical ward without being admitted to the ICU because they survived the acute decompensation but subsequently died from the progression of their hematological illness.

The baseline hematological characteristics are summarized in Table 1. The baseline comparison between alive and dead at hospital discharge is summarized in Table 2.

Patients who died had generally more severe systemic disease at first MET evaluation (SOFA 8.0 vs. 5.9 and MEWS 4.9 vs. 3.8) than those who survived at hospital discharge. Those patients also had longer hospitalization before MET referral (3 vs. 10 days) and sepsis was more likely the cause of the acute decompensation. No difference in age, sex, comorbidity (CCI), and prior performance status (ECOG) was found. Also, respiratory failure severity categorized as PaO_2_/FiO_2_ and cardiovascular failure defined as the need for pressor support before the MET visit were similar in the two groups. Neutropenia (Neutrophil count < 500 cells/µL) and pharmacological immunosuppression were also comparable. MET was alerted immediately after the onset of the symptoms prodromic of a critical illness (median of 0 days in both dead and alive patients) and no difference was evident between those two groups.

**Figure 2:**
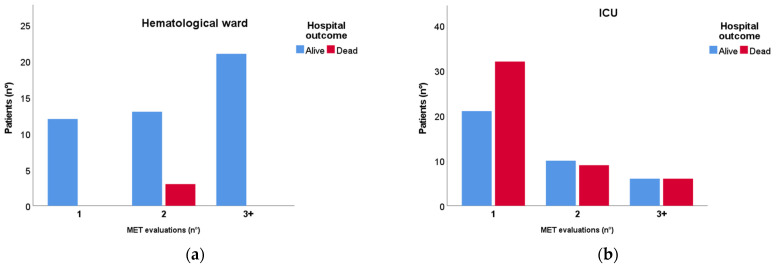
Mortality distribution between 1, 2, and 3 or more (3+) MET evaluations in patients treated exclusively in the hematological ward (**a**) and in those admitted to the ICU (**b**). MET: Medical emergency team; ICU: Intensive care unit.

Figure 2 represents the number of MET consults that each patient received before being considered stable enough to be treated without MET assistance (Panel A) or being too severe and therefore admitted to the ICU (Panel B). Three patients died without being admitted to the ICU as already described. Eighty-four patients were admitted to the ICU, 53 of which (63%) immediately after the first MET referral. Those who received more than a MET evaluation were admitted to the ICU for 1 day (IQR 0;2) after the first MET consult. No difference between the delay of admission of patients who died and those who survived was found (*p* = 0.214).

Overall, 65 (49%) patients received only 1 MET evaluation, 35 (26%) had 2 evaluations, and 33 (25%) had 3 or more evaluations. Twelve (9%) patients received only one MET consult and were stable enough to continue the cure in the hematological ward without the prolonged assistance of an intensivist (SOFA 5.0 ± 1.9, MEWS 2.3 ± 1.4, PaO_2_/FiO_2_ 255 ± 81). Fifty-three (40%) patients were so severe that they were immediately transferred to the ICU after the first MET consult. Those patients differed significantly from those who were re-evaluated by MET in terms of acute disease severity (SOFA, MEWS, and vital parameters such as respiratory rate, heart rate, blood pressure, and mental status were significantly more severe in the former group) as shown in Table 3. No difference in PaO_2_/FiO_2_, neutropenia and pharmacological immunosuppression was evident.

In the sub-population analysis of the 68 patients who received at least two MET consults, 43 (63%) received helmet CPAP support and 12 (18%) received amine support in the medical ward. Considering patients who received a CPAP trial in the hematological ward, no difference in the first MET evaluation was evident between those who were admitted to the ICU and those who successfully continued treatments in the medical ward considering PaO_2_/FiO_2_ value, SOFA, and MEWS (191 vs. 220 mmHg, *p* = 0.334; 6.2 vs. 6.0, *p* = 0.679; 3.6 vs. 3.4, *p* = 0.611, respectively). Similar results were obtained in those who received pressors (SOFA 8.4 vs. 8.3, *p* = 0.899; MEWS 4.4 vs. 4.7, *p* = 0.411). Moreover, 53% of the patients who received helmet CPAP support were admitted to the ICU (23 patients), while the ICU admission rate in those without a CPAP trial was 32% (*p* = 0.086). Patients admitted to the ICU with a prior helmet CPAP trial had a trend of higher mortality than those admitted without it (56.5 vs. 25%, *p* = 0.124).

Those receiving amine support had a similar ICU access rate than those who did not require cardiovascular support (41.7 vs. 46.4%; *p* = 0.764).

## 4. Discussion

In this monocentric retrospective analysis, we found that the MET selection of hematological critically ill patients able to complete an intensive treatment trial in the hematological ward is effective and safe. Reassuringly, we recorded a low mortality (6%) in the subgroup treated entirely in the hematological ward, which was not related to the acute decompensation but rather to further the progression of the hematological disease (Figure 2A). A relevant number of patients recovered from the critical episode in the hematological ward (37%), thereby avoiding a relocation to a less isolated environment and decreasing the demand for ICU beds. Furthermore, no increase in mortality was detected in patients initially treated by the MET in the hematological ward compared to those immediately transferred to the ICU (Figure 2B). Importantly, the time of admission was not associated with mortality (*p* = 0.214).

We performed a sub-population analysis of patients who received at least two MET evaluations. Therefore, we excluded the patients with a more severe acute illness (see Table 3) and, as such, immediately transferred to the ICU, and patients with milder conditions that did not require further MET interventions. Thus, we selected the patients who might benefit from a “critical care without wall” trial. Interestingly, at the beginning of the trial (for both helmet CPAP or cardiovascular support), it was not possible to estimate the subsequent necessity of ICU admission, either with clinical judgment or considering PaO_2_/FiO_2_ value, SOFA, and MEWS.

Remarkably, our data regarding the prognostic factors of hematological patients who develop a critical illness are consistent with those of the more recent literature [4,5,6,7]. The severity of the critical illness is confirmed to be predictive of mortality in our patients. SOFA and MEWS were effective tools for prognostication: Higher values of both are associated with mortality (Table 2) and immediate ICU admission (Table 3). Patients who died had a greater incidence of sepsis as the cause of the acute decompensation than those who survived, in line with the literature (6). Also, longer hospitalization prior to MET referral is associated with a worse prognosis; this aspect has also been previously discussed in the literature and is likely related to several factors such as frailty, prolonged bedridden condition, malnutrition, and hospital-acquired infection [18,19].

Apparently, mortality is not influenced by age, comorbidity (CCI), or prior performance status (ECOG). However, we must consider that our population was composed only of patients selected by a multidisciplinary team as eligible for ICU treatment. Excluding the “do not reanimate” patients may have flattened the difference in these parameters.

Neutropenia (Neutrophil count < 500 cells/µL) and pharmacological immunosuppression were not more frequent in patients who died than those who survived; these data are in line with the recent literature [20]. We were not able to analyze the baseline hematological disease in relation to mortality due to the wide variety of conditions and the subsequent low number of patients in each category (Table 1).

Finally, the analysis conducted on the specific procedure adopted in the medical ward highlighted that the patients who needed CPAP support had a higher ICU access rate and higher mortality, even if these data were not statistically significant (ICU access rate: 53 vs. 32, *p* = 0.086; in-hospital mortality: 56.5 vs. 25%, *p* = 0.124). The findings are consistent with those of a previous study demonstrating that respiratory events were independently associated with both ICU admission and hospital mortality [21]. This underlines the need for improving the management strategy of patients with acute respiratory failure, as onco-hematological conditions are adjunctive risk factors for ARDS [22]. Also, it has been previously suggested that early use of CPAP in the hematological ward could prevent evolution to acute lung injury requiring mechanical ventilation and ICU admission [23]. Hemodynamic impairment does not seem to be a prognostic factor since the patients who received a trial with pressors had a similar ICU access rate as those who did not require cardiovascular support (41.7 vs. 46.4%; *p* = 0.764). The analysis of mortality was not performed on patients with pharmacological cardiovascular support because of the low numerosity of the group.

This study has several limitations, mainly related to the timing of data collection: Data were collected only at the first and last MET evaluations, missing the ones in between. It would, hence, be quite an expected result to observe a deterioration in PaO_2_/FiO_2_, SOFA, and MEWS from the first to the last record in patients who were admitted to the ICU as opposed to those who continued the treatment in the ward. It would be interesting for further trials to consider collecting these parameters at fixed time points after the first evaluation, because the evolution of the acute illness and the response to the treatment may be powerful prognostic and, thus, strategy-guiding factors. Moreover, patients who were not immediately transferred to the ICU spent 1 day (IQR 0;2) between MET activation and ICU admission. We collected our data considering days as the time unit. However, due to the rapid evolution of the critical illness and the rather frequent MET re-evaluation (at least every 8 to 12 h), we suggest future researchers evaluate this time in hours, since even smaller delays may be effective in changing the prognosis. Moreover, this is a monocentric study conducted in a hospital where the MET is composed of intensivists and nurses with extensive clinical experience. Furthermore, the MET has been active in our hospital for several years and is therefore a finely structured service. The study replicability is therefore limited by the presence of a well-established service with experience and skills ranging across numerous medical specialties.

In conclusion, the MET organization described in this paper requires that its staff are exclusively dedicated to the specific service. Therefore, it necessitates more human resources than a conventional system in which the intensivist works most of the shift in the ICU and intervenes only if contacted. However, the MET uses resources already dedicated to a single patient, anticipating clinical deterioration and therefore likely reducing costs, although such an analysis is beyond the scope of this study. We are persuaded that this system guarantees the administration of intensive organ support therapies in a safe manner, saving intensive care resources in terms of nursing and ICU beds.

## 5. Conclusions

An intensive treatment trial in the medical ward provided with MET support on hematological patients who develop a critical illness may be effective in avoiding ICU admission. A prospective and multicentric trial, which would also include the evolution of the acute illness in the first hours after MET intervention, might be useful.

## Figures and Tables

**Table 1:** Hematological baseline characteristics. * Neutrophil count < 500 cells/µL.

	*N* = 133 (%)
Hematological diagnosis	
- Non-Hodgkin Lymphoma	38 (29)
- Acute myeloid leukemia	36 (27)
- Multiple Myeloma	17 (13)
- Acute lymphocytic leukemia	16 (12)
- Chronic lymphocytic leukemia	5 (4)
- Myelodysplasia	5 (4)
- Bone marrow aplasia	4 (3)
- Hodgkin Lymphoma	3 (2)
- Others	9 (7)
Disease state	
- Onset	55 (42)
- Complete remission	41 (31)
- Relapse < 1 year	5 (4)
- Relapse > 1 year	19 (14)
- Refractory disease	12 (9)
- Unknown	1 (1)
Bone marrow transplant	
- Autologous	5 (4)
- Allogenic	30 (23)
Graft-vs.-host disease	
- Acute	16 (12)
- Chronic	2 (2)
Neutropenia *	46 (35)
Ongoing chemotherapy	76 (57)
Pharmacological immunosuppression	62 (47)

**Table 2:** Population baseline comparison between outcomes at hospital discharge. CCI: Charlson comorbidity index; SOFA: Sequential organ failure assessment; MEWS: Modified early warning score; ICU: Intensive care unit; MET: Medical emergency team; ECOG: Eastern cooperative oncology group *: *p* < 0.05; ^†^: value recorded at first MET evaluation, £: Neutrophil count < 500 cells/µL.

	Alive (*N* = 83)	Dead (*N* = 50)	*p*-Value
Age—Years	56 ± 13	58 ± 12	0.395
Sex—Female no. (%)	32 (39)	19 (38)	0.949
Days of hospitalization before MET evaluation	3 (0; 13)	10 (0; 23)	0.032 *
Days between critical illness symptoms and MET evaluation—median (IQR)	0 (0; 1)	0 (0; 1)	0.509
CCI	5.3 ± 2.4	5.0 ± 2.0	0.407
SOFA score ^†^	5.9 ± 2.5	8.0 ± 2.6	<0.001 *
MEWS ^†^	3.8 ± 2.2	4.9 ± 2.3	0.007 *
ECOG performance status—median (IQR)	0 (0; 1)	0 (0; 1)	0.163
PaO_2_/FiO_2_—mmHg ^†^	241 ± 115	212 ± 113	0.201
Amine support—no. (%) ^†^	7 (8)	5 (10)	0.760
Neutropenia £—no. (%)	30 (36)	16 (32)	0.626
Pharmacological immunosuppression—no. (%)	35 (54)	27 (42)	0.185
Sepsis—no. (%)	30 (36)	28 (56)	0.025 *
ICU admission—no. (%)	37 (45)	47 (94)	<0.001 *

**Table 3:** Baseline comparison between patients immediately admitted to ICU and patients who received at least a second MET consult. SOFA: Sequential organ failure assessment; MEWS: Modified early warning score; ICU: Intensive care unit; MET: Medical emergency team; ECOG: Eastern cooperative oncology group *: *p* < 0.05; ^†^: value recorded at first MET evaluation.

	Immediately Admitted to ICU (*N* = 53)	2 or More MET Evaluations (*N* = 68)	*p*-Value
Respiratory rate >30 bpm ^†^—no. (%)	19 (36)	19 (28)	0.042 *
Heart rate >130 bpm ^†^—no. (%)	13 (25)	8 (12)	<0.001 *
Systolic blood pressure <70 mmHg ^†^—no. (%)	15 (28)	1 (2)	<0.001 *
Mental status alteration ^†^—no. (%)	12 (23)	4 (6)	0.010 *
SOFA score †	7.9 ± 2.9	6.0 ± 2.2	<0.001 *
MEWS †	5.6 ± 2.5	3.5 ± 1.6	<0.001 *
PaO_2_/FiO_2_—mmHg ^†^	213 ± 126	239 ± 108	0.252
Neutropenia—no. (%)	41 (77)	38 (56)	0.014 *
Pharmacological immunosuppression—no. (%)	23 (43)	32 (47)	0.688
Sepsis—no. (%)	28 (52)	28 (41)	0.202

## Data Availability

The data presented in this study are available upon request from the corresponding author. The data are not publicly available due to privacy restrictions.

## References

[1] Darmon M., Bourmaud A., Georges Q., Soares M., Jeon K., Oeyen S., Rhee C.K., Gruber P., Ostermann M., Hill Q.A. (2019). Changes in critically ill cancer patients’ short-term outcome over the last decades: Results of systematic review with meta-analysis on individual data. Intensive Care Med..

[2] Azoulay E., Mokart D., Pène F., Lambert J., Kouatchet A., Mayaux J., Vincent F., Nyunga M., Bruneel F., Laisne L.-M. (2013). Outcomes of critically ill patients with hematologic malignancies: Prospective multicenter data from France and Belgium—A groupe de recherche respiratoire en réanimation onco-hématologique study. J. Clin. Oncol..

[3] Denardo S.J., Oye R.K., Bellamy P.E. (1989). Efficacy of intensive care for bone marrow transplant patients with respiratory failure. Crit. Care Med..

[4] Kiehl M.G., Beutel G., Böll B., Buchheidt D., Forkert R., Fuhrmann V., Knöbl P., Kochanek M., Kroschinsky F., La Rosée P. (2018). Consensus statement for cancer patients requiring intensive care support. Ann. Hematol..

[5] Meert A.-P., Wittnebel S., Holbrechts S., Toffart A.-C., Lafitte J.-J., Piagnerelli M., Lemaitre F., Peyrony O., Calvel L., Lemaitre J. (2021). Critically ill cancer patient’s resuscitation: A Belgian/French societies’ consensus conference. Intensive Care Med..

[6] Taheri L., Anandanadesan R., de Lavallade H., Pagkalidou E., Pagliuca A., Mufti G., Auzinger G., Metaxa V. (2019). The role of a critical care outreach service in the management of patients with haematological malignancy. J. Intensive Care Soc..

[7] Rhodes A., Ferdinande P., Flaatten H., Guidet B., Metnitz P.G., Moreno R.P. (2012). The variability of critical care bed numbers in Europe. Intensive Care Med..

[8] Song J.-U., Suh G.Y., Park H.Y., Lim S.Y., Han S.G., Kang Y.R., Kwon O.J., Woo S., Jeon K. (2012). Early intervention on the outcomes in critically ill cancer patients admitted to intensive care units. Intensive Care Med..

[9] Bauer J., Brüggmann D., Klingelhöfer D., Maier W., Schwettmann L., Weiss D.J., Groneberg D.A. (2020). Access to intensive care in 14 European countries: A spatial analysis of intensive care need and capacity in the light of COVID-19. Intensive Care Med..

[10] Biard L., Darmon M., Lemiale V., Mokart D., Chevret S., Azoulay E., Resche-Rigon M. (2019). Center Effects in Hospital Mortality of Critically Ill Patients with Hematologic Malignancies. Crit. Care Med..

[11] Guidelines for intensive care unit admission, discharge, and triage (1999). Task Force of the American College of Critical Care Medicine, Society of Critical Care Medicine. Crit. Care Med..

[12] Blanch L., Abillama F.F., Amin P., Christian M., Joynt G.M., Myburgh J., Nates J.L., Pelosi P., Sprung C., Topeli A. (2016). Triage decisions for ICU admission: Report from the Task Force of the World Federation of Societies of Intensive and Critical Care Medicine. J. Crit. Care.

[13] Ramos J.G.R., Perondi B., Dias R.D., Miranda L.C., Cohen C., Carvalho C.R.R., Velasco I.T., Forte D.N. (2016). Development of an algorithm to aid triage decisions for intensive care unit admission: A clinical vignette and retrospective cohort study. Crit. Care.

[14] Hillman K.M. (2002). Critical care without walls. Curr. Opin. Crit. Care.

[15] Austin C.A., Hanzaker C.B., Stafford R.M., Mayer C., Culp L.R., Lin F.-C., Chang L. (2014). Utilization of rapid response resources and outcomes in a comprehensive cancer center. Crit. Care Med..

[16] Jung B., Daurat A., De Jong A., Chanques G., Mahul M., Monnin M., Molinari N., Jaber S. (2016). Rapid response team and hospital mortality in hospitalized patients. Intensive Care Med..

[17] De Jong A., Jung B., Daurat A., Chanques G., Mahul M., Monnin M., Molinari N., Jaber S. (2016). Effect of rapid response systems on hospital mortality: A systematic review and meta-analysis. Intensive Care Med..

[18] Park J., Lee Y.J., Hong S.-B., Jeon K., Moon J.Y., Kim J.S., Kang B.J., Ahn J.-J., Lee D.-H., Park J. (2021). The association between hospital length of stay before rapid response system activation and clinical outcomes: A retrospective multicenter cohort study. Respir. Res..

[19] Lee J., Shin Y., Choi E., Choi S., Son J., Jung Y.K., Hong S.-B. (2021). Impact of hospitalization duration before medical emergency team activation: A retrospective cohort study. PLoS ONE.

[20] Bouteloup M., Perinel S., Bourmaud A., Azoulay E., Mokart D., Darmon M. (2017). Outcomes in adult critically ill cancer patients with and without neutropenia: A systematic review and meta-analysis of the Groupe de Recherche en Réanimation Respiratoire du patient d’Onco-Hématologie (GRRR-OH). Oncotarget.

[21] Doukhan L., Bisbal M., Chow-Chine L., Sannini A., Brun J.P., Cambon S., Duong L.N., Faucher M., Mokart D. (2017). Respiratory events in ward are associated with later intensive care unit (ICU) admission and hospital mortality in onco-hematology patients not admitted to ICU after a first request. PLoS ONE.

[22] Rhee C.K., Kang J.Y., Kim Y.H., Kim J.W., Yoon H.K., Kim S.C., Kwon S.S., Kim Y.K., Kim K.H., Moon H.S. (2009). Risk factors for acute respiratory distress syndrome during neutropenia recovery in patients with hematologic malignancies. Crit. Care.

[23] Squadrone V., Massaia M., Bruno B., Marmont F., Falda M., Bagna C., Bertone S., Filippini C., Slutsky A.S., Vitolo U. (2010). Early CPAP prevents evolution of acute lung injury in patients with hematologic malignancy. Intensive Care Med..

